# Yeasts of Burden: Exploring the Mycobiome–Bacteriome of the Piglet GI Tract

**DOI:** 10.3389/fmicb.2019.02286

**Published:** 2019-10-08

**Authors:** Ann M. Arfken, Juli Foster Frey, Timothy G. Ramsay, Katie Lynn Summers

**Affiliations:** Animal Biosciences and Biotechnology Laboratory, United States Department of Agriculture–Agricultural Research Service, Beltsville, MD, United States

**Keywords:** mycobiome, bacteriome, microbiome, piglet, weaning, swine

## Abstract

Interactions between the bacteria and fungi in the gut microbiome can result in altered nutrition, pathogenicity of infection, and host development, making them a crucial component in host health. Associations between the mycobiome and bacteriome in the piglet gut, in the context of weaning, remain unknown. Weaning is a time of significant stress, dietary changes, microbial alterations, and a predisposition to infection. The loss of animal health and growth makes potential microbial interventions of interest to the swine industry. Recent studies have demonstrated the diversity and development of the microbiome in the gastrointestinal (GI) tract of piglets during weaning, resulting from the dietary and physiological changes. Despite these advances, the role of the mycobiota in piglet health and its contribution to overall microbiome development remains mostly unknown. In this study we investigated the bacteriome and the mycobiome after weaning in the GI tract organs and feces from 35-day old piglets. Following weaning, the α-diversity and amplicon sequence variants (ASVs) counts of the bacteriome increased, proximally to distally, from the stomach to the feces along the GI tract, while the mycobiome α-diversity and ASV counts were highest in the porcine stomach. β-diversity analyses show distinct clusters based on organ type in the bacteriome and mycobiome, but dispersion remained relatively constant in the mycobiome between organ/fecal sites. Bacteroidetes, Firmicutes, and Epsilonbacteraeota were the most abundant bacterial phyla present in the GI tract and feces based on mean taxonomic composition with high variation of composition found in the stomach. In the mycobiome, the dominant phyla were Ascomycota and Basidiomycota, and the stomach mycobiome did not demonstrate the same high level of variation observed in the bacteriome. Potential interactions between genera were found in the lower piglet GI bacteriome and mycobiome with positive correlations found between the fungus, *Kazachstania*, and several bacterial species, including *Lactobacillus*. *Aspergillus* demonstrated negative correlations with the short chain fatty acid-producing bacteria *Butyricoccus*, *Subdoligranulum*, and *Fusicatenibacter*. This study demonstrates the distinct colonization dynamics between fungi and bacteria in the GI tract and feces of piglets directly following weaning and the potential interactions of these microbes in the porcine gut ecosystem.

## Introduction

The microbiome plays a critical role in animal health through its ability to alter nutrition, physiology, immune system development and function, and through bacterial–fungal–host interactions. Fungi in the GI tract of piglets are ubiquitous members of the rare biosphere (Huffnagle and Noverr, 2013; Summers et al., 2019) and disruption of the mycobiome may result in disease, as it does in other species (Ott et al., 2008; Iliev et al., 2012; Mason et al., 2012a; Erb Downward et al., 2013; Li et al., 2014; Li Z. et al., 2019). Additionally, fungi affect gut community structure and function through genetic exchange, interactions with bacterial species, biofilm formation, secondary metabolite secretion, and potential antibiotic creation (Frey-Klett et al., 2011; Suhr and Hallen-Adams, 2015).

Recent studies have demonstrated that commensal fungi can alter host immunity during normal health, as well as modify the severity of some diseases (Mukherjee et al., 2015; Kureljusic et al., 2016; Weissenbacher-Lang et al., 2016; Iliev and Leonardi, 2017; Limon et al., 2017; Richard and Sokol, 2019). Fungi can alter host immune responses through direct and indirect actions in the GI tract via pattern recognition receptors (PRRs), the production of metabolites such as prostaglandin E_2_ (PGE_2_), and multiple virulence factors that assist in host tissue invasion and nutrient acquisition. PGE_2_ is an immunomodulator typically produced by immune cells that can also be secreted by some fungi, such as *Candida*, leading to extensive immune changes in humans (Kim et al., 2014). Further, studies suggest that commensal fungi may promote immune tolerance to commensal bacteria (Li X.V. et al., 2019). In the context of pigs, studies have documented the effect of mycotoxins, fungal secondary metabolites known for contaminating agricultural feed, on the immune response. Different mycotoxins have the ability to up- or down-regulate the immune response in pigs, and immunosuppressive mycotoxins may increase piglet susceptibility to infectious diseases (Pierron et al., 2016). Due to the known sensitivity of piglets to these fungal metabolites, future studies are vital to understand the role of commensal fungi in porcine health.

The weaning transition is a stressful time in a pig’s life and associated changes in the piglet gut microbiome can result in poor health and reduced growth performance, making it of critical interest to the swine industry (Campbell et al., 2013; Guevarra et al., 2018, 2019). Post-weaning diarrhea and susceptibility to opportunistic pathogens are common consequences of changes to the piglet gastrointestinal (GI) microbiome. Recently, studies have begun to elucidate the normal members of the microbiota in piglets, but details remain unknown as to interactions among members alter immune responses and promote growth performance. This information is necessary to identify potential alternative growth promotants as the use of antibiotics for growth promotion is banned in the United States. While studies have begun to show the importance of weaning and diet changes in the development of the GI microbiome (Bian et al., 2016; Han et al., 2017), the mycobiota remains a poorly understood, yet integral part of the gut ecosystem.

Members of the microbiota interact with each other within the host environment through a variety of means, including physical or chemical interactions, competition for resources or space, production of biofilms, or modulation of the surrounding environment (Krüger et al., 2019). For example, studies have demonstrated the ability of bacterial metabolites to directly inhibit *Candida* growth and colonization in the gut (Nguyen et al., 2011; Bulgasem et al., 2016) as well as the production of mycotoxins by bacteria residing within the fungal cytosol (Partida-Martinez and Hertweck, 2005). Bacteria can also indirectly inhibit fungal growth by activating different components of the immune system. One such example is the ability of lactobacilli to promote host resistance to gut colonization with *Candida* spp. through the activation of AhR, a transcription factor that stimulates the release of IL-22 (Kiss et al., 2011; Zelante et al., 2013; Lamas et al., 2016). In humans, *Candida albicans* can prevent the gut colonization of other fungal and bacterial pathogens (Tso et al., 2018) and *Aspergillus fumigatus* can inhibit *Pseudomonas aeruginosa* and alter the pro-inflammatory immune response in co-cultures (Reece et al., 2018). The microbial interplay in the piglet gut may significantly alter the growth and health of pigs long-term due to the numerous potential interactions between fungi and bacteria. Recent studies have demonstrated a link between certain fungal species and weight gain in other mammalian species (Mar Rodriguez et al., 2015), and while currently unknown, potential dietary intervention strategies for piglet weight gain is of great interest to industry (Sam et al., 2017). Previous work from our laboratory has shown that the dominant, post-weaning fungal species is *Kazachstania slooffiae*, but its role in animal health and development remains to be elucidated (Summers et al., 2019). We hypothesize that the bacteriome and mycobiome will significantly differ between organ sites. The current study investigated the microbiome and mycobiome in piglets 2 weeks post-weaning to evaluate the diversity, populations, and potential interactions between the bacterial and fungal members of the piglet GI tract and feces.

## Materials and Methods

### Animal Procedures

A 23 Large White × Landrace piglets from 3 litters (L.119 = 8 piglets, L.120 = 8 piglets, and L.126 = 7 piglets) were assessed from birth through day 35 of age and were weaned at day 21. Piglets were not provided with creep feed or milk replacer at any point throughout the experiment. The diet was formulated to meet the National Research Council estimate of nutrient requirements (Supplementary Table S1). From days 21–28, piglets received Nursery Diet 1 followed by Nursery Diet 2 from days 29–35. Piglets were evaluated daily for health and were given free access to feed and water; all piglets used in this study were observed to be healthy. No antibiotics, antifungals, or supplementary additives were administered to the piglets at any time during the experiment. On day 35 of age, piglets were humanely euthanized, and the GI tract was removed from the abdominal cavity and immediately dissected. Sections from the stomach, proximal duodenum, jejunum, distal ileum, cecum, distal colon, and feces were collected under sterile conditions and luminal contents removed with a PBS wash. Mucosal-associated microbial populations were investigated due to their proximity with the host and the potential to alter host tissue responses. Organ sections and feces were placed in sterile cryovials, flash frozen in liquid nitrogen, and stored at −80°C until further processing. Care and treatment of all pigs were approved by the USDA–ARS Institutional Animal Care and Use Committee of the Beltsville Agricultural Research Center.

### DNA Extraction and Sequencing

DNA was isolated from 0.25 g feces or organ sections using the MagAttract Power Microbiome Kit (Qiagen, Hilden, Germany) by the Microbial Systems Molecular Biology Laboratory at the University of Michigan. Cells were lysed to isolate DNA using mechanical bead beating for 20 total minutes with 20 frequency/second and extracted using magnetic bead technology according to the Qiagen protocol. The V4 region of the 16S rRNA-encoding gene was amplified from extracted DNA using the barcoded dual-index primers developed previously (Kozich et al., 2013). The ITS region was sequenced utilizing primers ITS3 (5′ GCATCGATGAAGAACGCAGC-3′) AQ3 and ITS4 (5′-TCCTCCGCTTATTGATATGC-3′) with the Illumina adaptor sequence added to the 5′ end (5′-TCGTCGGCAGCGTCAGATGTG TATAAGAGACAG—ITS3-3′) and (5′GCTTCGTGGGCTCGGAGATGTGTATAAG AGACAG—ITS4-3′). Both 16S and ITS regions were sequenced with the Illumina MiSeq Sequencing platform.

### Bacteriome (16S) and Mycobiome (ITS) Sequence Processing

#### Bacteria (16S)

Quality filtering, pairing, denoising, amplicon sequence variants (ASVs) determination, and chimera removal was conducted with the DADA2 plugin (Callahan et al., 2016) in QIIME2 v. 2019.4 (Caporaso et al., 2010). For quality trimming, paired-end sequences were truncated to 240 and 160 bp for forward and reverse reads, respectively, with an average median quality score of 34.8. Taxonomic classification of the ASVs was performed using the pretrained 16S 515F/806R from the Silva 132 database (Yilmaz et al., 2014). ASVs identified as Archaea, chloroplast, mitochondria, or unassigned were removed from further analysis.

#### Fungi (ITS)

Forward and reverse primers were removed from paired-end reads with cutadapt v 1.18 (Martin, 2011). QIIME2 plugin DADA2 was used to perform similar quality filtering and ASV identification described above for bacterial sequences. Because of the variable nature of fungal ITS sequencing length, however, no quality trimming was conducted on fungal sequences. Average median quality score was 35.9 and 32.3 for forward and reverse reads, respectively. Taxonomic classification was trained and conducted on fungal sequences using the UNITE (Koljalg et al., 2013) developer’s full-length ITS reference sequences in QIIME2. Fungal ASVs without a phylum or higher classification or those identified as unassigned were removed. Additional classification using BLAST^1^ was performed on removed sequences to confirm non-fungal origin.

Separate rarefaction curves for bacterial and fungal samples were produced using the vegan package (Oksanen et al., 2019) in R v 3.5.1^2^ and visualized in GraphPad Prism v 7 (La Jolla, CA, United States) to determine minimum sequencing depth. A cutoff of 5,000 sequences was determined for bacterial and fungal samples. Samples <5000 sequences were removed (bacteria, *n* = 30; fungus, *n* = 11). 130 bacterial and 149 fungal samples were selected for downstream analysis.

### Characterization of the Bacteriome and Mycobiome

Calculations of α-diversity were performed on rarefied (*n* = 5,000 sequences) bacterial and fungal samples using the phyloseq package (McMurdie and Holmes, 2013). Shannon diversity indices and observed ASVs were normalized using box cox and square root transformations, respectively. Satisfaction of normality was tested using the Shapiro–Wilk test. Differences between bacterial and fungal Shannon diversity and observed ASVs were determined using a linear mixed model with organ as the fixed effect and pig as the random effect using the lmer4 and lmerTest. Non-metric multidimensional scaling (NMDS) was conducted using the vegan package on log-transformed bacterial and fungal sequences using Bray–Curtis dissimilarity distances. To reduce potential ASV artifacts, ASVs with <1 sequence in ≤5.0% of samples were removed prior to analysis. NMDs plots were visualized using the ggplot2 package (Wickham, 2016). Pairwise comparisons of mean Bray–Curtis distances to group centroids was calculated using the permutational analysis of multivariate dispersion (PERMDISP) function in vegan and plotted in R. Due to similarities between organ bacteriomes and mycobiomes, samples were recategorized by GI region: duodenum, jejunum, and ileum samples were recategorized as “Upper GI,” cecum and colon were recategorized as “Lower GI,” and stomach and feces remained categorized as “Stomach” and “Feces,” respectively. For visualization purposes, relative abundances of taxa are presented as mean% value by litter for each GI tract region and feces.

### Correlation and Network Analyses of the Lower GI

For correlation analysis, samples were rarefied to their corresponding bacterial or fungal sample pair to account for sequencing depth differences between pairs while retaining similar community composition structure. Bacterial (*n* = 46) and fungal sample pairs (*n* = 46) were combined and ASVs were merged at the genus level. Genera found <30% of samples were removed to prevent degradation of correlation detection, which increases with increased numbers of 0 counts (Weiss et al., 2017). Correlations between fungus and bacteria were detected using the sparse correlations for composition (SparCC) python module (Friedman and Alm, 2012). Correlation values were visualized using the corrplot package in R. *P*-values were corrected for multiple comparisons using FDR. A corresponding network analysis of SparCC correlation coefficients was created using the igraph (Csardi and Nepusz, 2005) and the Sparse and Compositionally Robust Inference of Microbial Ecological Networks (Kurtz et al., 2015) R-packages. Only correlations with an absolute value ≥0.4 were plotted. Unless otherwise stated, all statistical tests were performed in R, *p*-values of <0.05 were considered significant, and errors are given as ±SE. All figures were created with GraphPad Prism 7, unless otherwise indicated.

## Results

### Composition and Diversity of the Bacteriome and Mycobiome in the Piglet GI Tract

To analyze the microbiota communities in the piglet GI tract, the V4 and ITS2 regions of the bacterial 16S rRNA and fungal ITS genes, respectively, were amplified and sequenced from feces and six organ sections (stomach, duodenum, jejunum, ileum, cecum, and colon) collected from 23 piglets, aged 35 days. A total of 8,363,058 bacterial and 6,670,225 fungal high quality sequences were obtained following the QIIME processing and filtering pipeline. Rarefaction curves showed that a minimum sampling depth of 5,000 sequences was sufficient to capture both bacterial and fungal diversity in organs and feces (Supplementary Figures S2A,B). After removal of samples with <5000 sequences, the number of bacterial and fungal samples was reduced to 130 and 149, respectively (Supplementary Tables S2A,B). A mean sequencing depth of 24,909 ± 1,448 and a total of 2383 ASVs were detected in bacterial samples, and a mean sequencing depth of 35,187 ± 1,585 and a total of 592 ASVs were detected in fungal samples.

**FIGURE 1:**
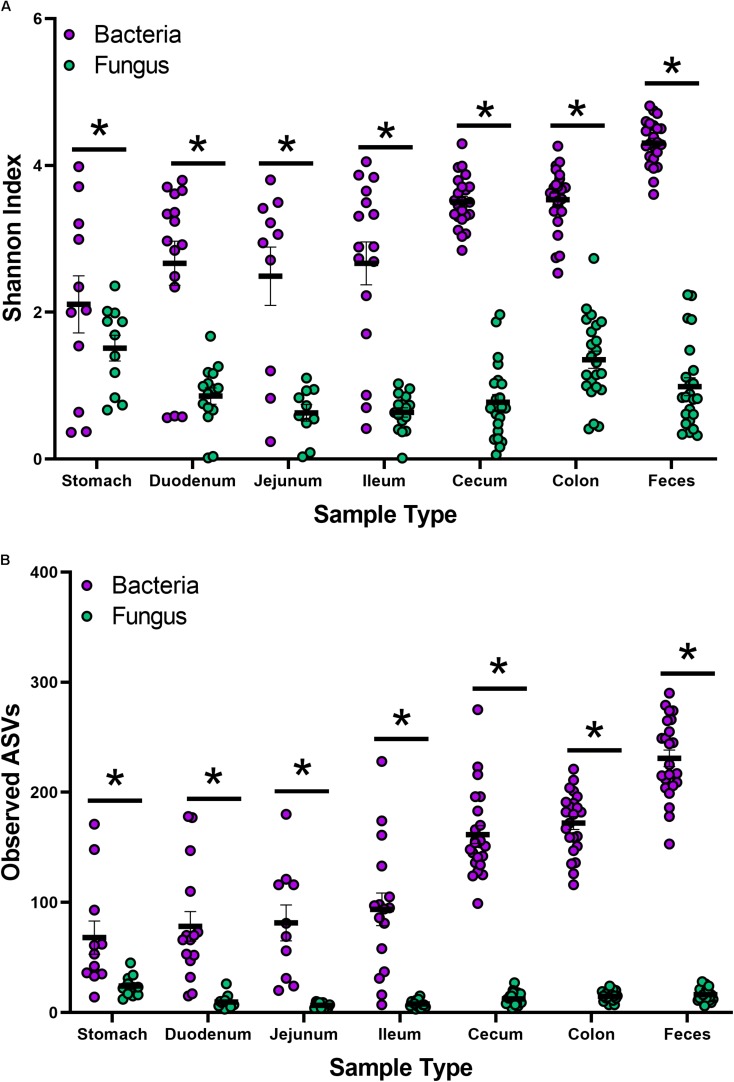
Alpha-diversity of the bacteriome and mycobiome in piglet GI organs. **(A)** Shannon diversity index values and **(B)** observed ASV counts for bacterial 16S rRNA and fungal ITS gene sequencing data by sample type. Linear mixed-models were performed to determine differences between bacterial and fungal indices by organ. Only samples with both bacterial 16S rRNA and fungal ITS gene sequencing data were plotted and analyzed. Significance indicated by ^∗^*p* < 0.001.

Indices for Shannon and observed ASVs were calculated to measure the α-diversity in the bacteriome and mycobiome (Figures 1A,B and Supplementary Tables S3A,B). In the bacteriome, the overall trend showed an increase in diversity and observed ASVs from the stomach to the feces along the GI tract. The mycobiome showed a different trend, with the stomach showing higher diversity and observed ASVs, followed by a decrease in diversity and observed ASVs in the duodenum, jejunum, and ileum and an increase in diversity and observed ASVs in the colon. Compared to the mycobiome, diversity and observed ASVs were significantly higher in the bacteriome (*p* < 0.001, Supplementary Tables S2A,B).

**FIGURE 2:**
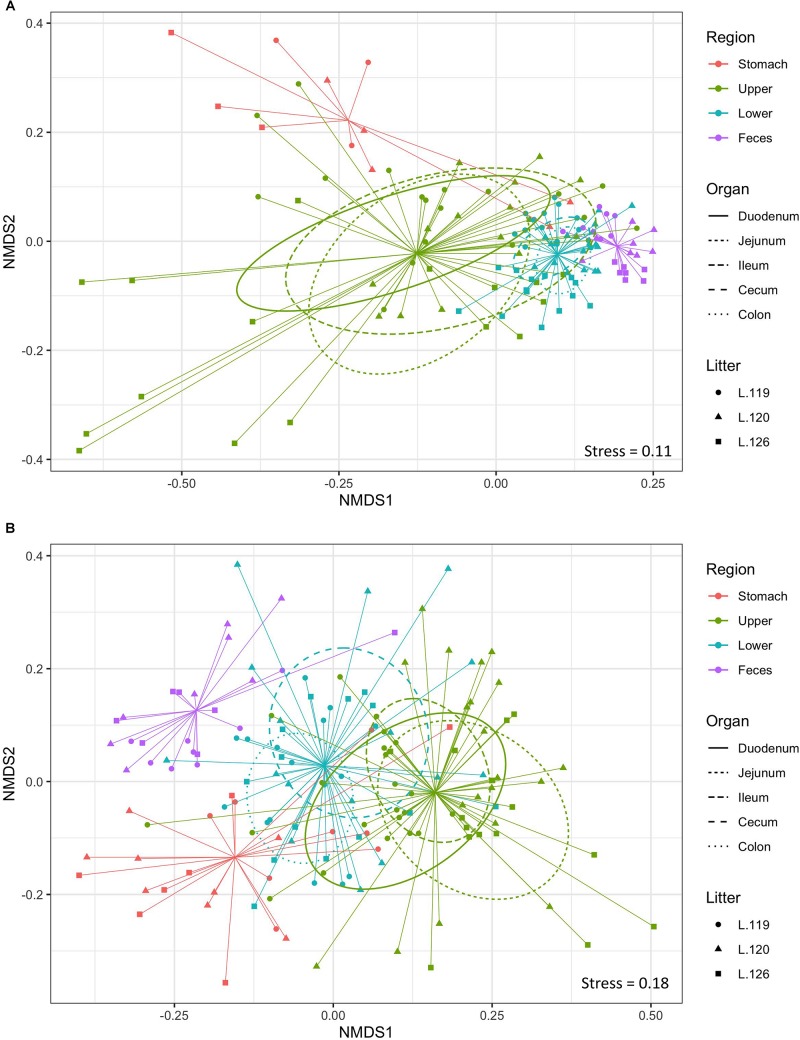
Beta-diversity of gastrointestinal tract organs and litters. Non-metric multidimensional scaling (NMDS) plot of β-diversity based on Bray–Curtis dissimilarities in the **(A)** bacteriome and **(B)** mycobiome of the piglet GI tract. Ellipses indicate 1 standard deviation from organ centroid and spiders are drawn to GI tract region centroid. Colors indicate GI tract region, symbols indicate litter, and ellipses line types indicate specific organs of the upper and lower GI.

**FIGURE 3:**
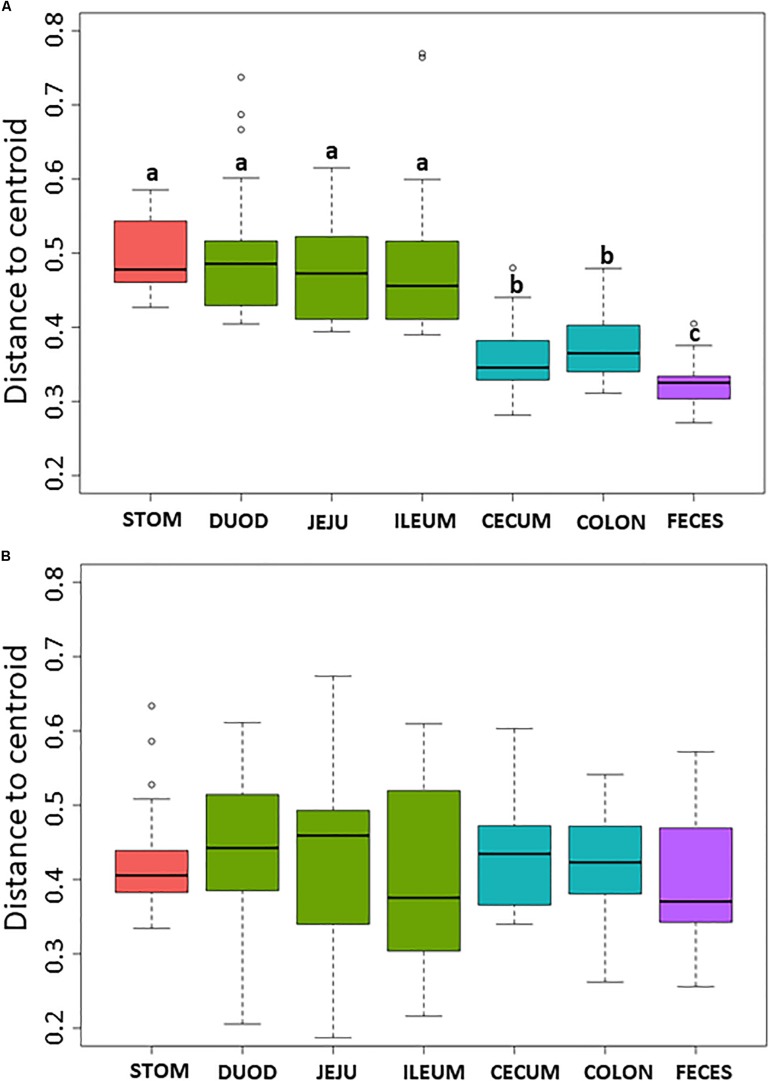
Box plot of pairwise distances between piglet organ centroids. Plots represent the median and interquartile range in the **(A)** bacteriome and **(B)** mycobiome. Colors indicate piglet GI tract region: red = stomach, green = upper GI, blue = lower GI, purple = feces. Differences between organ centroids were analyzed using permutational analysis of multivariate dispersion on Bray–Curtis dissimilarities with significance indicated by letters (*p* < 0.05).

Non-metric multidimensional scaling plot were used to visualize β-diversity between the different regions and organs of the piglet GI (Figures 2A,B). In both the bacteriome and mycobiome, stomach and feces showed distinct clusters from the other organs. The duodenum, jejunum, and ileum organs in the upper GI, and the cecum and colon in the lower GI had a high degree of overlap among their centroids within their respective GI tract region indicating similarities between the microbiota communities. Mean distances between group centroids (dispersion) for each organ were calculated using PERMDISP on Bray–Curtis dissimilarities (Supplementary Tables S4A,B). In the bacteriome, there was a significant decrease in dispersion from the stomach and upper GI tract to the lower GI tract and to the feces, signifying a larger amount of individual variation in the upper GI tract vs. the lower GI tract and feces (*p* < 0.05, Figure 3A and Supplementary Table S2A). This trend in dispersion directly contrasted with α-diversity, which showed an increase in observed ASVs and Shannon diversity from the stomach to the lower GI as shown previously (Figure 1A). The mycobiome showed no significant trends in dispersion and remained relatively similarly dispersed throughout the piglet GI tract and feces (*p* ≥ 0.05, Figure 3B, and Supplementary Table S2B).

**FIGURE 4:**
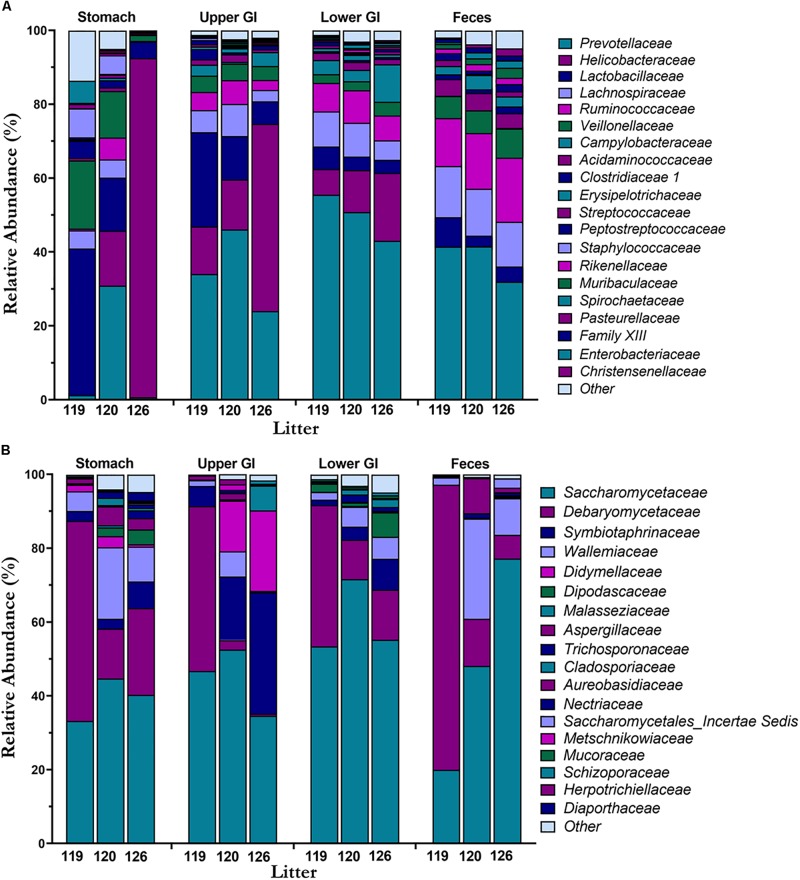
Taxonomic composition of the bacteriome and mycobiome in the piglet GI tract. Mean percent relative abundances by litter at the family level are shown for the most abundant members of the **(A)** microbiome and **(B)** mycobiome for each GI tract region.

Mean taxonomic composition by litter of bacterial and fungal families present in the piglet GI tract were compared across GI tract and feces (Figures 4A,B). In the bacteriome, the most abundant phyla (Supplementary Figure S2A) present in the GI tract and feces were Bacteroidetes (40.8 ± 1.9%), Firmicutes (37.2 ± 1.9%), and Epsilonbacteraeota (19.5 ± 2.5%) comprising >97% of the piglet bacteriome. Genera *Prevotella 9*, *Prevotella 1*, *Prevotellaceae* NK3B31 group, and *Alloprevotella* from family *Prevotellaceae* (38.2 ± 2.0%), *Helicobacter* from family *Helicobacteraceae* (17.4 ± 2.4%), *Lactobacillus* from family *Lactobacillaceae* (10.3 ± 1.4%), *Blautia* from family *Lachnospiraceae* (7.2 ± 0.4%), and *Veillonella f*rom family *Veillonellaceae* (5.2 ± 0.6%) were among the most abundant and prevalent genera and families in the bacteriome (Supplementary Figure S2C). In general, bacterial families *Helicobacteraceae* and *Lactobacillaceae* decreased from the stomach and upper GI to the lower GI and feces, while families *Prevotellaceae*, *Lachnospiraceae*, and *Ruminoccocaceae* increased along the GI tract and feces. Relative abundances of *Helicobacteraceae* in the feces were <1.0%. Of the different GI tract regions, the stomach showed high variation in taxonomic composition among litters, while the lower GI and feces showed relatively consistent taxa across litters. In the mycobiome, Ascomycota (90.7 ± 1.3%) and Basidiomycota (9.0 ± 1.2%) made up the dominant phyla (Supplementary Figure S2B). Genera *Kazachstania* (sp. *sloofiae*) from family *Saccharomycetaceae* (49.6 ± 2.8%), *Hyphopichia* from family *Debaryomycetaceae* (23.2 ± 2.3%), and Wallemia from family *Wallemiaceae* (6.3 ± 1.0%) were the dominant genera and families across all GI tract regions and feces (Figure 4B and Supplementary Figure S2D). *Symbiotaphrina* from family *Symbiotaphrinaceae* was dominant in the piglet GI tract organs (9.8 ± 2.0%) but was only found in 3 piglet feces samples at <0.1% abundance. In contrast to the stomach and feces bacteriome, the stomach mycobiome had relatively consistent taxa among the litters, while the feces mycobiome demonstrated a high degree of variation.

### Interactions Between the Bacteriome and Mycobiome in the Piglet Lower GI

**FIGURE 5:**
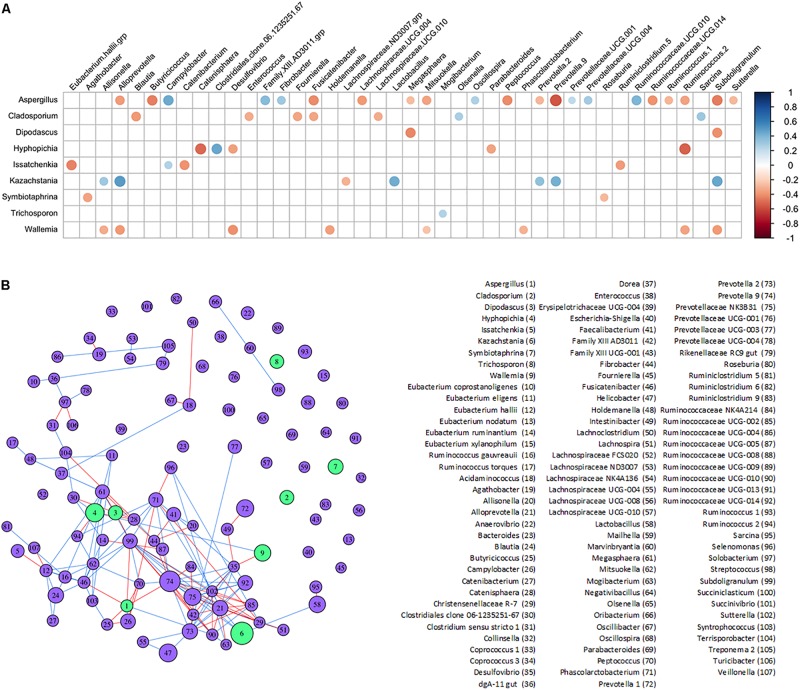
Inferred interactions between the bacteriome and mycobiome in the piglet lower GI tract. **(A)** SparCC correlation plot showing significant individual correlations between bacterial and fungal genera in the lower GI organs of the post-weaning piglet. Red circles indicate negative correlations and blue circles indicate positive correlations (*p* < 0.05 after FDR adjustments). The size of circles represents correlation strength while non-significant correlations are not shown. **(B)** SparCC correlation network between bacterial and fungal genera with plotted correlations with an absolute value ≥0.4. The edge color indicates sign of correlation: negative (red), positive (blue); node color indicates kingdom: bacteria (purple), fungus (green). The size of node is proportional to the mean centered-log ratio abundance for each genus.

Potential interactions between genera found in the lower piglet GI bacteriome and mycobiome were determined with SparCC correlations and a corresponding network analysis (Figures 5A,B and Supplementary Tables S5A,B). Fungi genus *Kazachstania* showed significant positive correlations with bacteria genera *Alloprevotella*, *Lactobacillus*, *Prevotella 9*, and *Subdoligranulum*. Fungi genera *Aspergillus*, *Cladosporium*, *Hyphopichia*, and *Wallemia* showed mostly negative correlations with other bacteria genera. *Aspergillus*, in particular, showed predominantly negative correlations with short chain fatty acid-producing bacteria such as *Butyricicoccus, Subdoligranulum* and *Fusicatenibacter.* Fungi genera *Diopadascus*, *Symbiotaphrina*, and *Trichosporon* did not show correlations with other fungi or bacteria. Additionally, there were no strong correlations among any of the other fungi genera identified within the piglet gut mycobiome.

## Discussion

Fungi, in addition to bacteria, are important members and contributors of the microbiome, and recognition of their role is an essential step forward in elucidating the dynamics of the GI environment. Of the limited gut studies examining bacteria and fungi together, none to our knowledge have explored the interaction of the bacteriome–mycobiome of the piglet GI tract. Our study characterized and compared the mucosal-associated bacteriome and mycobiome of 23 healthy piglets, aged 35 days, from 3 litters along different organs of the GI tract and feces. The lower GI tract, which included the cecum and colon, was further evaluated to assess differences in predicted interactions between the bacteriome and mycobiome and determine associations between bacterial and fungal genera. This research is a critical first step in revealing the complex interactions, including health and growth, promoted by the fungi in the piglet gut during weaning.

While studies investigating the microbiota have become common, extensive studies of the mycobiota have been limited due to a lack of technologies, databases, and consensus in techniques (Li X.V. et al., 2019; Richard and Sokol, 2019). Recently, Li X.V. et al. (2019) reviewed studies characterizing the mycobiome of different human sites and disease states. Despite significant recent advances, methodologies continue to lack consensus for DNA isolation, primer design, sequencing, databases, and analysis of fungal species. Previous work in our laboratory aimed to determine which techniques and fungal primers were effective in studying the piglet mycobiome (Summers et al., 2019). Lower estimated diversity in the mycobiome compared to the bacteriome has been observed in human stool (Nash et al., 2017), piglet feces (Summers et al., 2019), and settling dust in pig farms (White et al., 2019). While there is no established consensus on what constitutes a healthy gut mycobiome, it is widely accepted that fungi are less abundant and demonstrate less diversity than bacteria in the human gut, comprising roughly 0.1% of the microbiome based on shotgun metagenome sequencing (Qin et al., 2010; Huffnagle and Noverr, 2013). In this study, compared to the bacteriome, estimated overall diversity and observed ASVs for all 6 GI organs (stomach, duodenum, jejunum, ileum, cecum, colon) and feces were significantly lower in the mycobiome (Figures 1A,B and Supplementary Figures S1A,B).

Within the bacteriome, there was an increasing trend in α-diversity along the GI tract from the stomach to the colon, with highest α-diversity found in the feces, coupled with a decreasing trend in dispersion (β-diversity) among the organs (Figure 1A and Supplementary Tables S3, S4). These trends are consistent with those seen by Crespo-Piazuelo et al. (2018), which characterized the bacteriomes of 120 day old pigs along the GI tract gradient from the duodenum to the distal colon. In general, the stomach and upper GI tract (duodenum, jejunum, and ileum) host fewer microorganisms than the lower GI tract (colon and cecum) due to shorter retention times for adherence to tissue or mucus (Donaldson et al., 2016) lower pH, and higher concentrations of bile acids (Mackie et al., 1999; Walter and Ley, 2011). The harsher environment of the stomach and upper GI may subsequently select for a smaller number of colonizing bacterial species resulting in reduced diversity. The stomach and organs associated with the upper GI bacteriome also demonstrated higher levels of dispersion than the lower GI and feces, indicating a greater level of individual variation among piglets. Unlike the lower GI, the stomach and upper GI are exposed to new and exogenous bacteria ingested with food particles (Donaldson et al., 2016). The stomach, in particular, serves to block ingested microbes from passing to the intestine (Martinsen et al., 2005). Despite identical piglet diets, individual variation was seen in the stomach and upper GI, potentially due to the amount and timing of the piglet’s meal. Other potential factors, including host immunity or fungal interactions, may influence bacterial variation in the upper GI of the piglet as the small intestine plays a critical role in the development of mucosal and systemic tolerance toward microbes (Villmones et al., 2018).

Much less is known about diversity trends in the gut mycobiome. Unlike the bacteriome, the mycobiome did not follow the same general linear increase in α-diversity along the GI tract (Figure 1B). Instead, the stomach mycobiome had the highest mean diversity, followed by the colon. The average gastric pH of 6-month old pigs fed *ad libitum* is 4.4, although this level can vary among individuals or timing of meals (Merchant et al., 2011). Compared to many bacteria, fungi are more acid tolerant. Many fungi have adaptive strategies to respond to low pH environments, and some fungi like *Aspergillus* sp. actively lower the surrounding pH of their environment (Vylkova, 2017). This suggests that the high diversity of the stomach mycobiome may be due to the greater survivability of fungi in highly acidic environments, as well as potentially less competition from bacteria for resources compared to the rest of the GI tract. Individual variation in the mycobiome remained relatively similar along the GI tract based on dispersion estimates (Figure 2B). Unlike the bacteriome, however, there was no reduction in dispersion in the lower GI or feces, and individual variation remained comparable to the upper GI bacteriome. Some studies have suggested that most fungi found in the GI tract are transient via environmental or dietary sources, and are unable to colonize or inhabit the gut long-term (Suhr and Hallen-Adams, 2015; Raimondi et al., 2019). The suspected temporary nature of some fungi in the piglet GI tract, as well as the genetic and immunity factors that affect the bacteriome, may all play a role in the relatively high level of individual variation in the mycobiome. Despite high individual variation within each organ mycobiome, distinct clusters were found for each GI tract region depicted in the NMDS (Figure 2B), indicating that fungal distribution along the GI tract is not random and may indicate different GI environmental niche effects on the mycobiome.

In both the bacteriome and mycobiome, there were dominant taxa throughout most of the piglet GI tract and feces (Figures 3A,B). In the bacteriome, Bacteroidetes, Firmicutes, and Epsilonbactereota were the dominant phyla, consistent with previous studies investigating the pig GI tract (Zhao et al., 2015; Kelly et al., 2017; Crespo-Piazuelo et al., 2018; Zhang et al., 2018). In previous studies, Proteobacteria was considered a dominant phylum, but recently Epsilonbactereota was reclassified as a separate phylum from Proteobacteria (Waite et al., 2017) and Epsilonbactereota was a more dominant phylum in our dataset. In general, there were increases in *Prevotellaceae*, *Lachnospiraceae*, and *Ruminoccocaceae* from the stomach to the lower GI tract, corresponding with a decrease in *Lactobacillaceae* and *Helicobacteraceae*. Many of these taxa shifts may be attributed to changing environmental conditions that occur along the GI tract. Measured dissolved oxygen levels undergo a dramatic reduction from the duodenum in the upper GI to the cecum of the lower GI (Hillman et al., 1993), as well as a reduction in pH, an increase in resistant starches (Flint et al., 2012), and slower peristalsis times (Walter and Ley, 2011). Members of *Helicobacteraceae* and *Lactobacillaceae* are tolerant of bile acids and oxygen (De Boever and Verstraete, 1999; Okoli et al., 2007; Yasuda et al., 2015), and are able to adhere firmly to the surface of the small intestine (Donaldson et al., 2016) making them suitable for colonizing the upper GI tract. In comparison, *Prevotellaceae*, *Lachnospiraceae*, and *Ruminoccoccaceae* are oxygen-sensitive and are likely more competitive in the lower GI due to their ability to degrade complex carbohydrates (Arumugam et al., 2011; Flint et al., 2012; Liu et al., 2012).

The dominant phyla in the piglet GI tract and feces mycobiome were Ascomycota and Basidiomycota, which are similar to those found in human mycobiome gut studies (Hoffmann et al., 2013; Nash et al., 2017; Raimondi et al., 2019). Unlike human studies, however, commonly found yeasts from genera *Candida* and *Saccharomyces* (Hallen-Adams and Suhr, 2017; Sam et al., 2017), were either absent or found at <1% relative abundance in our study samples. Instead, the dominant yeast throughout the piglet GI tract and feces was identified as *Kazachstania* (sp. *sloofiae*). *K. sloofiae* has been previously identified from different parts of the healthy pig GI tract (Uden and Carmo-Sousa, 1962; Urubschurov and Janczyk, 2011) and feces (Summers et al., 2019), and has been shown to establish quickly in the gut of piglets based on fecal analysis (Urubschurov et al., 2015; Urubschurov et al., 2017). Urubschurov et al. (2017) determined *K. sloofiae* may be responsible for maintaining piglet health by producing peptides, vitamin C, and formic acid in the piglet GI tract. Other dominant fungi genera found throughout the piglet GI tract and feces included *Hyphopichia* (sp. *burtonii*) and *Wallemia*. In contrast to *K. sloofiae*, both of these fungi are likely non-colonizing, transient fungi of the piglet GI tract. *Hyphopichia burtonii*, a commonly isolated yeast from corn, wheat, and rice (Kurtzman, 2011), has an estimated maximum growth temperature of 37°C (Burgain et al., 2015), and is unlikely to thrive at the internal temperature of a pigs at around 38.7–40°C. *Wallemia*, commonly isolated from food sources as well as agricultural dust, is also unlikely to reside in the piglet GI environment due to its extremophilic and xerophilic nature (Zajc and Gunde-Cimerman, 2018).

Complex interactions between bacteria and fungi also occur within the gut. Several significant correlations were found between bacterial and fungal genera in the lower piglet GI tract, suggesting potential bacteriome–mycobiome relationships (Figures 5A,B). The dominant fungal genus in the GI tract of humans is *Candida* and several species have been found to interact directly with bacterial species like *Lactobacillus* (Mason et al., 2012b; Allonsius et al., 2017; Hallen-Adams and Suhr, 2017; Rossoni et al., 2018). *Kazachstania* was strongly and positively correlated with *Lactobacillus* in the lower GI of our piglets and as *Kazachstania* is genetically similar to *Candida* (Kurtzman et al., 2005), it may be a potential porcine analog to *Candida* in the guts of humans. Future studies will be needed to clarify its role in pig gut health and homeostasis, as well as its potential to act as an opportunistic pathogen. A positive correlation between *Kazachstania* and *Lactobacillus* was also found in piglet feces in Urubschurov et al. (2011) using culture methods and PCR-DGGE techniques. In co-cultures of *Lactobacillus* and some yeasts, *Lactobacillus* released organic acids that lower the surrounding pH and promoted yeast growth; these yeasts are then stimulated by *Lactobacillus* to release essential nutrients and vitamins utilized by *Lactobacillus* (Stadie et al., 2013). A similar mutualistic relationship may exist between the dominant yeast *Kazachstania* in the piglet GI tract and *Lactobacillus*.

A strong positive correlation was also observed between *Kazachstania* and *Prevotella* 2 and *Prevotella* 9 genera. It has been hypothesized from observed positive associations between *Candida* yeasts and *Prevotella*, that *Candida* and *Prevotella* are involved in a mutualistic relationship regarding the degradation and fermentation of complex carbohydrates in the human gut (Hoffmann et al., 2013). In the piglet gut, *Kazachstania* may fulfill the role of *Candida* in the potential *Candida-Prevotella* link to starch metabolism. The corresponding network plot of the bacteriome–mycobiome community correlations showed no interactions between *Cladosporium*, *Symbiotaphrina*, and *Trichosporon* and other microbiota of the bacteriome–mycobiome. Unlike the *Kazachstania*-bacterial potential relationship, these fungi may be truly transient in the lower piglet GI and pass through without interacting with the gut microbial community. Interestingly, *Hyphopichia* and *Wallemia* showed mostly negative correlations with several gut bacteria. While these fungi are also thought to be non-colonizing microbiota of the gut as mentioned previously, they may still have an impact on the gut bacteriome during passage or may be capable of establishing themselves in the lower gut environment. One interesting finding in the lower gut of piglets was the negative association between short chain fatty acid-producing bacteria and *Aspergillus*. In humans, *Aspergillus* can exacerbate allergic responses in farm workers and is a well-documented pathogen. Aspergillosis is less common in pigs but has been documented as a rare cause of porcine abortions (Eustis et al., 1981; Todd et al., 1985; Sabino et al., 2012; Li Z. et al., 2019). *Aspergillus* has been documented to directly interact with bacterial species such as *Stenotrophomonas* and *Pseudomonas* and may play a critical role in disease severity (Schroeckh et al., 2009; Melloul et al., 2018; Briard et al., 2019). The negative association of *Aspergillus* with SCFA-producing bacteria, typically associated with beneficial gut health (Baxter et al., 2019; Peirce and Alvina, 2019) could be explained by the change in *Aspergillus* spp. behavior following sodium butyrate exposure (Philip et al., 1963).

In this study, we provided a comprehensive overview of the bacteria and fungi present along the piglet GI tract and feces in healthy piglets post-weaning, as well as new insight into potential interactions between the microbiome and mycobiome. The taxonomy and diversity of the mycobiome, in addition to the microbiome, demonstrated distinct differences in diversity between the bacterial and fungal members of the gut. In addition, fungal commensals, such as *Candida* spp., from the human gut were lacking in the pig. Potential interactions in the porcine gut show that bacteria may be acting in a beneficial way with the fungus, *Kazachstania*, and through negative interactions with *Aspergillus*. Further exploration of these significant correlations in the piglet gut will provide a greater understanding of the relationships that exist between the bacteriome and mycobiome that may potentially alter piglet growth and health.

## Data Availability Statement

The datasets generated for this study can be found using the Accession number PRJNA558038: https://www.ncbi.nlm.nih.gov/bioproject/PRJNA558038.

## Ethics Statement

The animal study was reviewed and approved by the USDA–ARS Institutional Animal Care and Use Committee of the Beltsville Agricultural Research Center.

## Author Contributions

AA and KS contributed to the conception and design of the study, and wrote the first draft of the manuscript. AA performed the statistical analysis. All authors performed the animal research and handling, contributed to manuscript revision, read, and approved the submitted version.

## Conflict of Interest

The authors declare that the research was conducted in the absence of any commercial or financial relationships that could be construed as a potential conflict of interest.

## Footnotes

1 https://blast.ncbi.nlm.nih.gov

2 https://www.R-project.org

## Supplementary Material

FIGURE S1 Rarefaction curves of mean observed ASVs in piglet organs and feces in **(A)** bacteria and **(B)** fungi. Error bars represent ±SE.

FIGURE S2 Taxonomic composition of the bacteriome and mycobiome in the piglet GI tract. Mean percent relative abundances by litter are shown for the top taxa at the **(A)** bacterial phylum, **(B)** fungal phylum, **(C)** bacterial genus, and **(D)** fungal genus level for each GI tract organ.

TABLE S1 Composition of diet for weaned piglets.

TABLE S2 Distribution of bacterial and fungal samples by sample type and litter.

TABLE S3 Summary results of **(A)** Shannon diversity and **(B)** observed ASV differences between bacteria and fungi in the piglet GI tract.

TABLE S4 Summary results of dispersion comparisons in the **(A)** bacteriome and **(B)** mycobiome along the piglet GI tract.

TABLE S5 Correlation **(A)** matrix and **(B)** FDR adjusted *p*-values between bacteria and fungi in the piglet lower GI tract.

## References

[B1] Allonsius C. N. van den Broek M. F. L. De Boeck I. Kiekens S. Oerlemans E. F. M. Kiekens F. (2017). Interplay between *Lactobacillus rhamnosus* gg and candida and the involvement of exopolysaccharides. *Microb. Biotechnol.* 10 1753–1763. 10.1111/1751-7915.12799 28772020 PMC5658588

[B2] Arumugam M. Raes J. Pelletier E. Le Paslier D. Yamada T. Mende D. R. (2011). Enterotypes of the human gut microbiome. *Nature* 473 174–180. 10.1038/nature09944 21508958 PMC3728647

[B3] Baxter N. T. Schmidt A. W. Venkataraman A. Kim K. S. Waldron C. Schmidt T. M. (2019). Dynamics of human gut microbiota and short-chain fatty acids in response to dietary interventions with three fermentable fibers. *mBio* 10:e02566-18. 10.1128/mBio.02566-18 30696735 PMC6355990

[B4] Bian G. Ma S. Zhu Z. Su Y. Zoetendal E. G. Mackie R. (2016). Age, introduction of solid feed and weaning are more important determinants of gut bacterial succession in piglets than breed and nursing mother as revealed by a reciprocal cross-fostering model. *Environ. Microbiol.* 18 1566–1577. 10.1111/1462-2920.13272 26940746

[B5] Briard B. Mislin G. L. A. Latge J. P. Beauvais A. (2019). Interactions between aspergillus fumigatus and pulmonary bacteria: current state of the field, new data, and future perspective. *J. Fungi* 5:48. 10.3390/jof5020048 31212791 PMC6617096

[B6] Bulgasem B. Y. Lani M. N. Hassan Z. Wan Yusoff W. M. Fnaish S. G. (2016). Antifungal activity of lactic acid bacteria strains isolated from natural honey against pathogenic candida species. *Mycobiology* 44 302–309. 10.5941/MYCO.2016.44.4.302 28154488 PMC5287163

[B7] Burgain A. Bensoussan M. Dantigny P. (2015). Validation of a predictive model for the growth of chalk yeasts on bread. *Int. J. Food Microbiol.* 204 47–54. 10.1016/j.ijfoodmicro.2015.03.026 25847185

[B8] Callahan B. J. McMurdie P. J. Rosen M. J. Han A. W. Johnson A. J. Holmes S. P. (2016). DADA2: High-resolution sample inference from Illumina amplicon data. *Nat. Methods* 13 581–583. 10.1038/nmeth.3869 27214047 PMC4927377

[B9] Campbell J. M. Crenshaw J. D. Polo J. (2013). The biological stress of early weaned piglets. *J. Anim. Sci. Biotechnol.* 4:19. 10.1186/2049-1891-4-19 23631414 PMC3651348

[B10] Caporaso J. G. Kuczynski J. Stombaugh J. Bittinger K. Bushman F. D. Costello E. K. (2010). QIIME allows analysis of high-throughput community sequencing data. *Nat. Methods* 7 335–336. 20383131 10.1038/nmeth.f.303 PMC3156573

[B11] Crespo-Piazuelo D. Estelle J. Revilla M. Criado-Mesas L. Ramayo-Caldas Y. Ovilo C. (2018). Characterization of bacterial microbiota compositions along the intestinal tract in pigs and their interactions and functions. *Sci. Rep.* 8:12727. 10.1038/s41598-018-30932-6 30143657 PMC6109158

[B12] Csardi G. Nepusz T. (2005). The igraph software package for complex network research. *InterJ. Complex Syst.* 1695 1–9.

[B13] De Boever P. Verstraete W. (1999). Bile salt deconjugation by *Lactobacillus plantarum* 80 and its implication for bacterial toxicity. *J. Appl. Microbiol.* 87 345–352. 10540235 10.1046/j.1365-2672.1999.00019.x

[B14] Donaldson G. P. Lee S. M. Mazmanian S. K. (2016). Gut biogeography of the bacterial microbiota. *Nat. Rev. Microbiol.* 14 20–32. 10.1038/nrmicro3552 26499895 PMC4837114

[B15] Erb Downward J. R. Falkowski N. R. Mason K. L. Muraglia R. Huffnagle G. B. (2013). Modulation of post-antibiotic bacterial community reassembly and host response by *Candida albicans*. *Sci. Rep.* 3:2191. 10.1038/srep02191 23846617 PMC3709164

[B16] Eustis S. L. Kirkbride C. A. Gates C. Haley L. D. (1981). Porcine abortions associated with fungi, actinomycetes, and *Rhodococcus* sp. *Vet. Pathol.* 18 608–613. 10.1177/030098588101800505 7281459

[B17] Flint H. J. Scott K. P. Duncan S. H. Louis P. Forano E. (2012). Microbial degradation of complex carbohydrates in the gut. *Gut Microb.* 3 289–306. 22572875 10.4161/gmic.19897 PMC3463488

[B18] Frey-Klett P. Burlinson P. Deveau A. Barret M. Tarkka M. Sarniguet A. (2011). Bacterial-fungal interactions: hyphens between agricultural, clinical, environmental, and food microbiologists. *Microbiol. Mol. Biol. Rev.* 75 583–609. 10.1128/MMBR.00020-11 22126995 PMC3232736

[B19] Friedman J. Alm E. J. (2012). Inferring correlation networks from genomic survey data. *PLoS Comput. Biol.* 8:e1002687. 10.1371/journal.pcbi.1002687 23028285 PMC3447976

[B20] Guevarra R. B. Hong S. H. Cho J. H. Kim B. R. Shin J. Lee J. H. (2018). The dynamics of the piglet gut microbiome during the weaning transition in association with health and nutrition. *J. Anim. Sci. Biotechnol.* 9:54. 10.1186/s40104-018-0269-6 30069307 PMC6065057

[B21] Guevarra R. B. Lee J. H. Lee S. H. Seok M. J. Kim D. W. Kang B. N. (2019). Piglet gut microbial shifts early in life: causes and effects. *J. Anim. Sci. Biotechnol.* 10:1. 10.1186/s40104-018-0308-3 30651985 PMC6330741

[B22] Hallen-Adams H. E. Suhr M. J. (2017). Fungi in the healthy human gastrointestinal tract. *Virulence* 8 352–358. 10.1080/21505594.2016.1247140 27736307 PMC5411236

[B23] Han G. G. Lee J. Y. Jin G. D. Park J. Choi Y. H. Chae B. J. (2017). Evaluating the association between body weight and the intestinal microbiota of weaned piglets via 16S rRNA sequencing. *Appl. Microbiol. Biotechnol.* 101 5903–5911. 28523395 10.1007/s00253-017-8304-7

[B24] Hillman K. Whyte A. L. Stewart C. S. (1993). Dissolved oxygen in the porcine gastrointesitnal tract. *Lett. Appl. Microbiol*. 16 299–302.

[B25] Hoffmann C. Dollive S. Grunberg S. Chen J. Li H. Wu G. D. (2013). Archaea and fungi of the human gut microbiome: correlations with diet and bacterial residents. *PLoS One* 8:e66019. 10.1371/journal.pone.0066019 23799070 PMC3684604

[B26] Huffnagle G. B. Noverr M. C. (2013). The emerging world of the fungal microbiome. *Trends Microbiol.* 21 334–341. 10.1016/j.tim.2013.04.002 23685069 PMC3708484

[B27] Iliev I. D. Funari V. A. Taylor K. D. Nguyen Q. Reyes C. N. Strom S. P. (2012). Interactions between commensal fungi and the C-type lectin receptor Dectin-1 influence colitis. *Science* 336 1314–1317. 10.1126/science.1221789 22674328 PMC3432565

[B28] Iliev I. D. Leonardi I. (2017). Fungal dysbiosis: immunity and interactions at mucosal barriers. *Nat. Rev. Immunol.* 17 635–646. 28604735 10.1038/nri.2017.55 PMC5724762

[B29] Kelly J. Daly K. Moran A. W. Ryan S. Bravo D. Shirazi-Beechey S. P. (2017). Composition and diversity of mucosa-associated microbiota along the entire length of the pig gastrointestinal tract; dietary influences. *Environ. Microbiol.* 19 1425–1438. 10.1111/1462-2920.13619 27871148

[B30] Kim Y. G. Udayanga K. G. Totsuka N. Weinberg J. B. Nunez G. Shibuya A. (2014). Gut dysbiosis promotes M2 macrophage polarization and allergic airway inflammation via fungi-induced PGE(2). *Cell Host Microb.* 15 95–102. 10.1016/j.chom.2013.12.010 PMC3957200 24439901

[B31] Kiss E. A. Vonarbourg C. Kopfmann S. Hobeika E. Finke D. Esser C. (2011). Natural aryl hydrocarbon receptor ligands control organogenesis of intestinal lymphoid follicles. *Science* 334 1561–1565. 10.1126/science.1214914 22033518

[B32] Koljalg U. Nilsson R. H. Abarenkov K. Tedersoo L. Taylor A. F. Bahram M. (2013). Towards a unified paradigm for sequence-based identification of fungi. *Mol. Ecol.* 22 5271–5277. 10.1111/mec.12481 24112409

[B33] Kozich J. J. Westcott S. L. Baxter N. T. Highlander S. K. Schloss P. D. (2013). Development of a dual-index sequencing strategy and curation pipeline for analyzing amplicon sequence data on the MiSeq Illumina sequencing platform. *Appl. Environ. Microbiol.* 79 5112–5120. 10.1128/AEM.01043-13 23793624 PMC3753973

[B34] Kureljusic B. Weissenbacher-Lang C. Nedorost N. Stixenberger D. Weissenbock H. (2016). Association between *Pneumocystis* spp. and co-infections with *Bordetella bronchiseptica*, *Mycoplasma hyopneumoniae* and *Pasteurella multocida* in Austrian pigs with pneumonia. *Vet J* 207 177–179. 10.1016/j.tvjl.2015.11.003 26654847

[B35] Krüger W. Vielreicher S. Kapitan M. Jacobsen I. D. Niemiec M. J. (2019). Fungal-bacterial interactions in health and disease. *Pathogens* 8:70. 10.3390/pathogens8020070 31117285 PMC6630686

[B36] Kurtz Z. D. Muller C. L. Miraldi E. R. Littman D. R. Blaser M. J. Bonneau R. A. (2015). Sparse and compositionally robust inference of microbial ecological networks. *PLoS Comput. Biol.* 11:e1004226. 10.1371/journal.pcbi.1004226 25950956 PMC4423992

[B37] Kurtzman C. P. (2011). Phylogeny of the ascomycetous yeasts and the renaming of *Pichia anomala* to Wickerhamomyces anomalus. *Antonie Van Leeuwenhoek* 99 13–23. 10.1007/s10482-010-9505-6 20838888

[B38] Kurtzman C. P. Robnett C. J. Ward J. M. Brayton C. Gorelick P. Walsh T. J. (2005). Multigene phylogenetic analysis of pathogenic candida species in the *Kazachstania* (*Arxiozyma*) *telluris* complex and description of their ascosporic states as *Kazachstania bovina* sp. nov., *K. heterogenica* sp. nov., *K. pintolopesii* sp. nov., and *K. slooffiae* sp. nov. *J. Clin. Microbiol.* 43 101–111. 15634957 10.1128/JCM.43.1.101-111.2005 PMC540161

[B39] Lamas B. Richard M. L. Leducq V. Pham H. P. Michel M. L. Da Costa G. (2016). CARD9 impacts colitis by altering gut microbiota metabolism of tryptophan into aryl hydrocarbon receptor ligands. *Nat. Med.* 22 598–605. 10.1038/nm.4102 27158904 PMC5087285

[B40] Li Q. Wang C. Tang C. He Q. Li N. Li J. (2014). Dysbiosis of gut fungal microbiota is associated with mucosal inflammation in Crohn’s disease. *J. Clin. Gastroenterol.* 48 513–523. 10.1097/MCG.0000000000000035 24275714 PMC4059552

[B41] Li X. V. Leonardi I. Iliev I. D. (2019). Gut mycobiota in immunity and inflammatory disease. *Immunity* 50 1365–1379. 31216461 10.1016/j.immuni.2019.05.023 PMC6585451

[B42] Li Z. Lu G. Meng G. (2019). Pathogenic fungal infection in the lung. *Front. Immunol.* 10:1524. 10.3389/fimmu.2019.01524 31333658 PMC6616198

[B43] Limon J. J. Skalski J. H. Underhill D. M. (2017). Commensal fungi in health and disease. *Cell Host Microb.* 22 156–165. 10.1016/j.chom.2017.07.002 28799901 PMC5573128

[B44] Liu H. Ivarsson E. Dicksved J. Lundh T. Lindberg J. E. (2012). Inclusion of chicory (*Cichorium intybus* L.) in pigs’ diets affects the intestinal microenvironment and the gut microbiota. *Appl. Environ. Microbiol.* 78 4102–4109. 10.1128/AEM.07702-11 22492453 PMC3370552

[B45] Mackie R. I. Sghir A. Gaskins H. R. (1999). Developmental microbial ecology of the neonatal gastrointestinal tract. *Am. J. Clin. Nutr.* 69 1035S–1045S. 10.1093/ajcn/69.5.1035s 10232646

[B46] Mar Rodriguez M. Perez D. Javier Chaves F. Esteve E. Marin-Garcia P. Xifra G. (2015). Obesity changes the human gut mycobiome. *Sci. Rep.* 5:14600. 10.1038/srep14600 26455903 PMC4600977

[B47] Martin M. (2011). Cutadapt removes adaptor sequences from high-throughput sequencing reads. *EMBnet J.* 17 10–12.

[B48] Martinsen T. C. Bergh K. Waldum H. L. (2005). Gastric juice: a barrier against infectious diseases. *Basic Clin. Pharmacol. Toxicol.* 96 94–102. 15679471 10.1111/j.1742-7843.2005.pto960202.x

[B49] Mason K. L. Erb Downward J. R. Falkowski N. R. Young V. B. Kao J. Y. Huffnagle G. B. (2012a). Interplay between the gastric bacterial microbiota and *Candida albicans* during postantibiotic recolonization and gastritis. *Infect. Immun.* 80 150–158. 10.1128/IAI.05162-11 21986629 PMC3255670

[B50] Mason K. L. Erb Downward J. R. Mason K. D. Falkowski N. R. Eaton K. A. Kao J. Y. (2012b). *Candida albicans* and bacterial microbiota interactions in the cecum during recolonization following broad-spectrum antibiotic therapy. *Infect. Immun.* 80 3371–3380. 10.1128/IAI.00449-12 22778094 PMC3457555

[B51] McMurdie P. J. Holmes S. (2013). phyloseq: an R package for reproducible interactive analysis and graphics of microbiome census data. *PLoS One* 8:e61217. 10.1371/journal.pone.0061217 23630581 PMC3632530

[B52] Melloul E. Roisin L. Durieux M. F. Woerther P. L. Jenot D. Risco V. (2018). Interactions of *Aspergillus fumigatus* and *Stenotrophomonas maltophilia* in an *in vitro* Mixed Biofilm Model: Does the strain matter? *Front. Microbiol.* 9:2850. 10.3389/fmicb.2018.02850 PMC6277776 30542331

[B53] Merchant H. A. McConnell E. L. Liu F. Ramaswamy C. Kulkarni R. P. Basit A. W. (2011). Assessment of gastrointestinal pH, fluid and lymphoid tissue in the guinea pig, rabbit and pig, and implications for their use in drug development. *Eur. J. Pharm. Sci.* 42 3–10. 10.1016/j.ejps.2010.09.019 20932902

[B54] Mukherjee P. K. Sendid B. Hoarau G. Colombel J. F. Poulain D. Ghannoum M. A. (2015). Mycobiota in gastrointestinal diseases. *Nat. Rev. Gastroenterol. Hepatol.* 12 77–87. 10.1038/nrgastro.2014.188 25385227

[B55] Nash A. K. Auchtung T. A. Wong M. C. Smith D. P. Gesell J. R. Ross M. C. (2017). The gut mycobiome of the human microbiome project healthy cohort. *Microbiome* 5:153. 10.1186/s40168-017-0373-4 29178920 PMC5702186

[B56] Nguyen L. N. Lopes L. C. Cordero R. J. Nosanchuk J. D. (2011). Sodium butyrate inhibits pathogenic yeast growth and enhances the functions of macrophages. *J. Antimicrob. Chemother.* 66 2573–2580. 10.1093/jac/dkr358 21911344

[B57] Okoli A. S. Wadstrom T. Mendz G. L. (2007). Bioinformatic study of bile responses in Campylobacterales. *FEMS Immunol. Med. Microbiol* 49 101–123. 17266717 10.1111/j.1574-695X.2006.00194.x

[B58] Oksanen J. Guillaume Blanchet F. Friendly M. Kindt R. Legendre P. McGlinn D. (2019). *vegan: Community Ecology Package.* Available at: https://cran.r-project.org (accessed July 05, 2019).

[B59] Ott S. J. Kuhbacher T. Musfeldt M. Rosenstiel P. Hellmig S. Rehman A. (2008). Fungi and inflammatory bowel diseases: alterations of composition and diversity. *Scand. J. Gastroenterol.* 43 831–841. 18584522 10.1080/00365520801935434

[B60] Partida-Martinez L. P. Hertweck C. (2005). Pathogenic fungus harbours endosymbiotic bacteria for toxin production. *Nature* 437 884–888. 16208371 10.1038/nature03997

[B61] Peirce J. M. Alvina K. (2019). The role of inflammation and the gut microbiome in depression and anxiety. *J. Neurosci. Res*. 97 1223–1241. 10.1002/jnr.24476 31144383

[B62] Philip E. T. Hall A. N. Walker T. K. (1963). Some effects of sodium n-butyrate on the behavior of aspergillus niger growing in a glucose medium. *Arch. Biochem. Biophys.* 102 238–241. 14061728 10.1016/0003-9861(63)90176-0

[B63] Pierron A. Alassane-Kpembi I. Oswald I. P. (2016). Impact of two mycotoxins deoxynivalenol and fumonisin on pig intestinal health. *Porcine Health Manag.* 2:21. 10.1186/s40813-016-0041-2 28405447 PMC5382503

[B64] Qin J. Li R. Raes J. Arumugam M. Burgdorf K. S. Manichanh C. (2010). A human gut microbial gene catalogue established by metagenomic sequencing. *Nature* 464 59–65. 10.1038/nature08821 20203603 PMC3779803

[B65] Raimondi S. Amaretti A. Gozzoli C. Simone M. Righini L. Candeliere F. (2019). Longitudinal survey of fungi in the human gut: its profiling, phenotyping, and colonization. *Front. Microbiol.* 10:1575 10.3389/fmicb.2019.01575 PMC6636193 31354669

[B66] Reece E. Doyle S. Greally P. Renwick J. McClean S. (2018). *Aspergillus fumigatus* Inhibits *Pseudomonas aeruginosa* in Co-culture: implications of a mutually antagonistic relationship on virulence and inflammation in the CF airway. *Front. Microbiol.* 9:1205. 10.3389/fmicb.2018.01205 29922270 PMC5996130

[B67] Richard M. L. Sokol H. (2019). The gut mycobiota: insights into analysis, environmental interactions and role in gastrointestinal diseases. *Nat. Rev. Gastroenterol. Hepatol.* 16 331–345. 10.1038/s41575-019-0121-2 30824884

[B68] Rossoni R. D. de Barros P. P. de Alvarenga J. A. Ribeiro F. C. Velloso M. D. S. Fuchs B. B. (2018). Antifungal activity of clinical *Lactobacillus* strains against *Candida albicans* biofilms: identification of potential probiotic candidates to prevent oral candidiasis. *Biofouling* 34 212–225. 10.1080/08927014.2018.1425402 29380647

[B69] Sabino R. Faisca V. M. Carolino E. Verissimo C. Viegas C. (2012). Occupational exposure to *Aspergillus* by swine and poultry farm workers in Portugal. *J. Toxicol. Environ. Health A* 75 1381–1391. 10.1080/15287394.2012.721170 23095156

[B70] Sam Q. H. Chang M. W. Chai L. Y. (2017). The fungal mycobiome and its interaction with gut bacteria in the host. *Int. J. Mol. Sci.* 18:E330. 10.3390/ijms18020330 28165395 PMC5343866

[B71] Schroeckh V. Scherlach K. Nutzmann H. W. Shelest E. Schmidt-Heck W. Schuemann J. (2009). Intimate bacterial-fungal interaction triggers biosynthesis of archetypal polyketides in *Aspergillus nidulans*. *Proc. Natl. Acad. Sci. U.S.A.* 106 14558–14563. 10.1073/pnas.0901870106 19666480 PMC2732885

[B72] Stadie J. Gulitz A. Ehrmann M. A. Vogel R. F. (2013). Metabolic activity and symbiotic interactions of lactic acid bacteria and yeasts isolated from water kefir. *Food Microbiol.* 35 92–98. 10.1016/j.fm.2013.03.009 23664259

[B73] Suhr M. J. Hallen-Adams H. E. (2015). The human gut mycobiome: pitfalls and potentials–a mycologist’s perspective. *Mycologia* 107 1057–1073. 26354806 10.3852/15-147

[B74] Summers K. L. Frey J. F. Ramsay T. G. Arfken A. M. (2019). The piglet mycobiome during the weaning transition: a pilot study1. *J. Anim. Sci.* 97 2889–2900. 10.1093/jas/skz182 31136650 PMC6606507

[B75] Todd J. N. Wells G. A. Davie J. (1985). Mycotic abortion in the pig. *Vet. Rec.* 116 350. 10.1136/vr.116.13.350 4002544

[B76] Tso G. H. W. Reales-Calderon A. J. Tan A. S. M. Sem X. Le G. T. T. Tan T. G. (2018). Experimental evolution of a fungal pathogen into a gut symbiont. *Science* 362 589–595. 10.1126/science.aat0537 30385579

[B77] Uden N. V. Carmo-Sousa L. D. (1962). On the intestinal yeast flora of free living hippopotami (*Hipopotamus amphibius*), wart hogs (*Phacochoerus aethiopicus*) and bush pigs (*Potamochoerus choeropotamus*). *Antonie Van Leeuwenhoek* 28 73–77. 13923439 10.1007/BF02538723

[B78] Urubschurov V. Busing K. Freyer G. Herlemann D. P. Souffrant W. B. Zeyner A. (2017). New insights into the role of the porcine intestinal yeast, *Kazachstania slooffiae*, in intestinal environment of weaned piglets. *FEMS Microbiol. Ecol*, 93:fiw245. 10.1093/femsec/fiw245 27940642

[B79] Urubschurov V. Busing K. Janczyk P. Souffrant W. B. Zeyner A. (2015). Development and evaluation of qPCR assay for quantitation of *Kazachstania slooffiae* and total yeasts occurring in the porcine gut. *Curr. Microbiol.* 71 373–381. 10.1007/s00284-015-0862-2 26134536

[B80] Urubschurov V. Janczyk C. (2011). “Biodiversity of yeasts in the gastrointestinal ecosystem with emphasis on its importance for the host,” in *The Dynamical Processes of Biodiversity - Case Studies of Evolution and Spatial Distribution*, eds Grillo O. Venora G. (Rijeka: INTECH), 277–302. 10.5772/24108

[B81] Urubschurov V. Janczyk P. Souffrant W. B. Freyer G. Zeyner A. (2011). Establishment of intestinal microbiota with focus on yeasts of unweaned and weaned piglets kept under different farm conditions. *FEMS Microbiol. Ecol.* 77 493–502. 10.1111/j.1574-6941.2011.01129.x 21569062

[B82] Villmones H. C. Haug E. S. Ulvestad E. Grude N. Stenstad T. Halland A. (2018). Species level description of the human ileal bacterial microbiota. *Sci. Rep.* 8:4736. 10.1038/s41598-018-23198-5 29549283 PMC5856834

[B83] Vylkova S. (2017). Environmental pH modulation by pathogenic fungi as a strategy to conquer the host. *PLoS Pathog.* 13:e1006149. 10.1371/journal.ppat.1006149 28231317 PMC5322887

[B84] Waite D. W. Vanwonterghem I. Rinke C. Parks D. H. Zhang Y. Takai K. (2017). Comparative genomic analysis of the class Epsilonproteobacteria and proposed reclassification to Epsilonbacteraeota (phyl. nov.). *Front. Microbiol.* 8:682 10.3389/fmicb.2017.00682 PMC5401914 28484436

[B85] Walter J. Ley R. (2011). The human gut microbiome: ecology and recent evolutionary changes. *Annu. Rev. Microbiol.* 65 411–429. 21682646 10.1146/annurev-micro-090110-102830

[B86] Weiss S. Xu Z. Z. Peddada S. Amir A. Bittinger K. Gonzalez A. (2017). Normalization and microbial differential abundance strategies depend upon data characteristics. *Microbiome* 5:27. 10.1186/s40168-017-0237-y 28253908 PMC5335496

[B87] Weissenbacher-Lang C. Kureljusic B. Nedorost N. Matula B. Schiessl W. Stixenberger D. (2016). Retrospective analysis of bacterial and viral co-infections in *Pneumocystis* spp. positive lung samples of austrian pigs with pneumonia. *PLoS One* 11:e0158479. 10.1371/journal.pone.0158479 27428002 PMC4948769

[B88] White J. K. Nielsen J. L. Madsen A. M. (2019). Microbial species and biodiversity in settling dust within and between pig farms. *Environ. Res.* 171 558–567. 10.1016/j.envres.2019.01.008 30771719

[B89] Wickham H. (2016). *ggplot2: Elegant Graphics for Data Analysis.* New York, NY: Springer International Publishing.

[B90] Yasuda K. Oh K. Ren B. Tickle T. L. Franzosa E. A. Wachtman L. M. (2015). Biogeography of the intestinal mucosal and lumenal microbiome in the rhesus macaque. *Cell Host Microb.* 17 385–391. 10.1016/j.chom.2015.01.015 25732063 PMC4369771

[B91] Yilmaz P. Parfrey L. W. Yarza P. Gerken J. Pruesse E. Quast C. (2014). The SILVA and all-species living tree project (LTP) taxonomic frameworks. *Nucleic Acids Res.* 42 D643–D648. 10.1093/nar/gkt1209 24293649 PMC3965112

[B92] Zajc J. Gunde-Cimerman N. (2018). The genus wallemia-from contamination of food to health threat. *Microorganisms* 6:E46. 10.3390/microorganisms6020046 29883408 PMC6027281

[B93] Zelante T. Iannitti R. G. Cunha C. De Luca A. Giovannini G. Pieraccini G. (2013). Tryptophan catabolites from microbiota engage aryl hydrocarbon receptor and balance mucosal reactivity via interleukin-22. *Immunity* 39 372–385. 10.1016/j.immuni.2013.08.003 23973224

[B94] Zhang X. Li Z. Yang H. Liu D. Cai G. Li G. (2018). Novel transgenic pigs with enhanced growth and reduced environmental impact. *eLife* 7:e34286. 10.7554/eLife.34286 29784082 PMC5963925

[B95] Zhao W. Wang Y. Liu S. Huang J. Zhai Z. He C. (2015). The dynamic distribution of porcine microbiota across different ages and gastrointestinal tract segments. *PLoS One* 10:e0117441. 10.1371/journal.pone.0117441 25688558 PMC4331431

