# Supplementary figures and images for: Yeasts of Burden: Exploring the Mycobiome–Bacteriome of the Piglet GI Tract
Source: Front Microbiol. 2019 Oct 8;10:2286. doi: 10.3389/fmicb.2019.02286 (PMC6792466; doi:10.3389/fmicb.2019.02286)

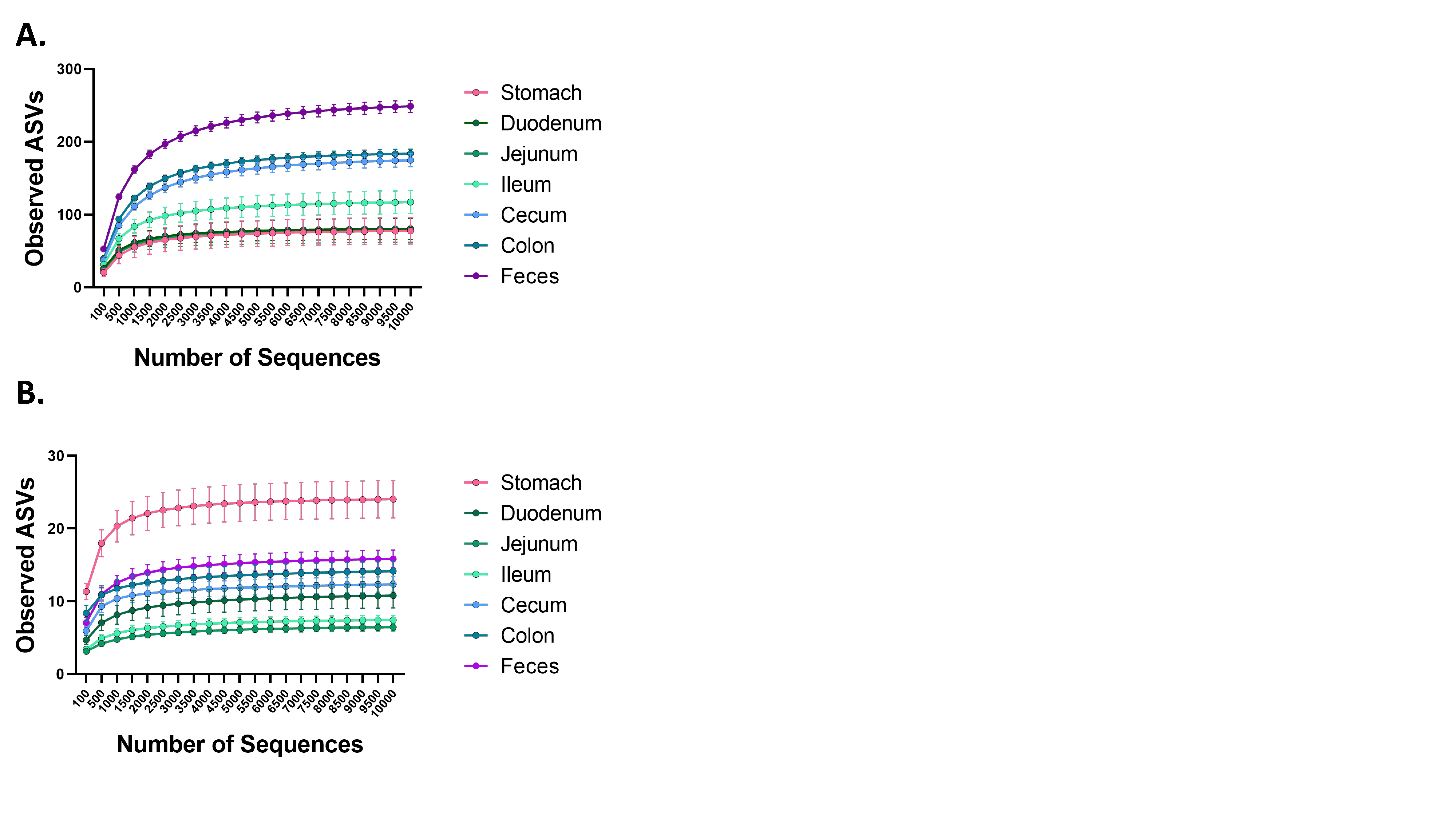

Supplement: FIGURE S1 — Rarefaction curves of mean observed ASVs in piglet organs and feces in (A) bacteria and (B) fungi. Error bars represent ±SE. [file Image_1.tiff]

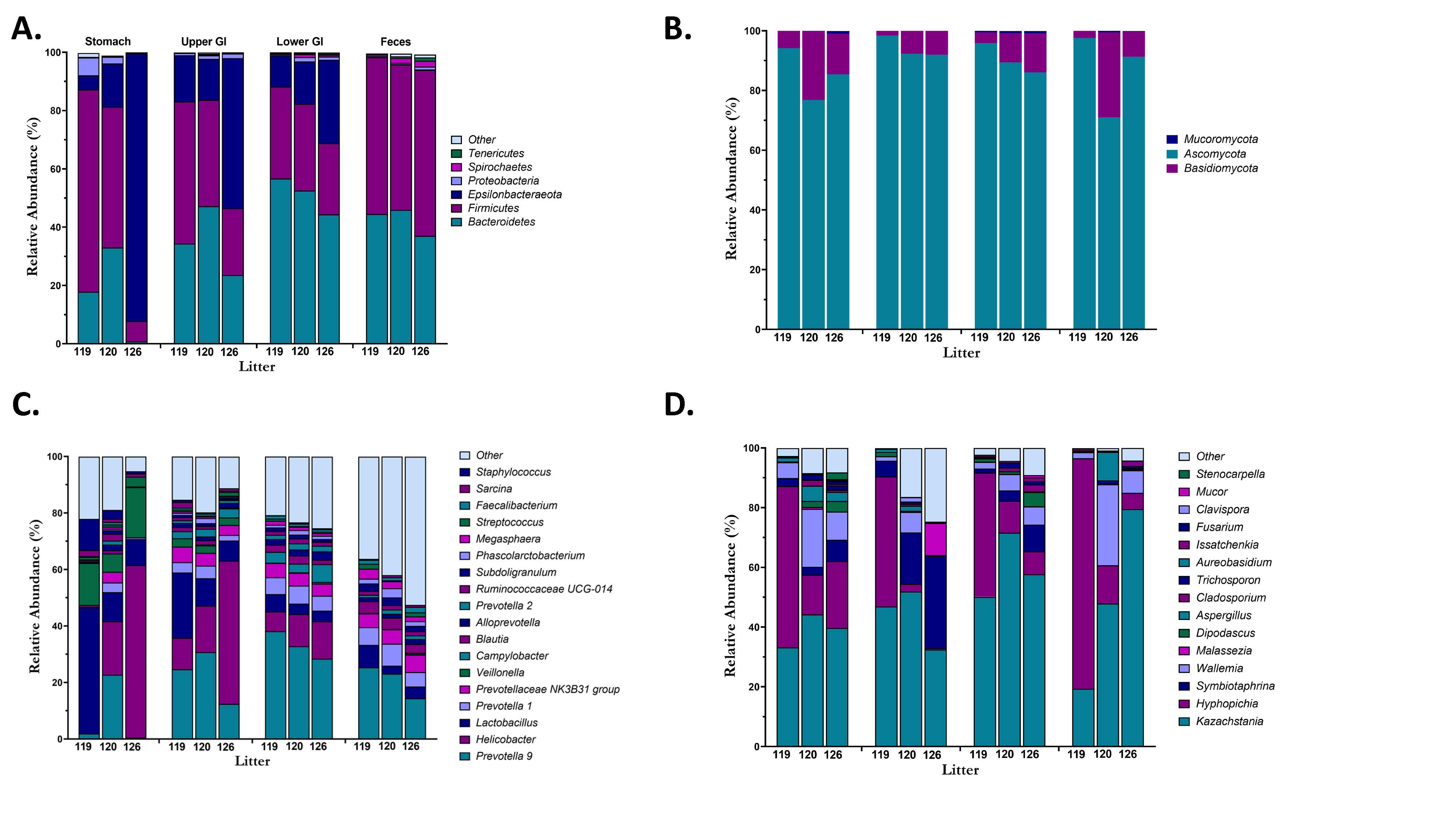

Supplement: FIGURE S2 — Taxonomic composition of the bacteriome and mycobiome in the piglet GI tract. Mean percent relative abundances by litter are shown for the top taxa at the (A) bacterial phylum, (B) fungal phylum, (C) bacterial genus, and (D) fungal genus level for each GI tract organ. [file Image_2.jpg]
